# Surgery As a Trigger for Incident Venous Thromboembolism: Results from a Population-Based Case-Crossover Study

**DOI:** 10.1055/a-2159-9957

**Published:** 2023-09-20

**Authors:** Dana Meknas, Sigrid K. Brækkan, John-Bjarne Hansen, Vânia M. Morelli

**Affiliations:** 1 Department of Clinical Medicine, Thrombosis Research Group, UiT—The Arctic University of Norway, Tromsø, Norway; 2 Department of Orthopedic Surgery, University Hospital of North Norway, Tromsø, Norway; 3 Division of Internal Medicine, Thrombosis Research Center, University Hospital of North Norway, Tromsø, Norway

**Keywords:** case-crossover study, venous thromboembolism, risk factor, surgery, immobilization, infection

## Abstract

**Background** Surgery is a major transient risk factor for venous thromboembolism (VTE). However, the impact of major surgery as a VTE trigger has been scarcely investigated using a case-crossover design.

**Aim** To investigate the role of major surgery as a trigger for incident VTE in a population-based case-crossover study while adjusting for other concomitant VTE triggers.

**Methods** We conducted a case-crossover study with 531 cancer-free VTE cases derived from the Tromsø Study cohort. Triggers were registered during the 90 days before a VTE event (hazard period) and in four preceding 90-day control periods. Conditional logistic regression was used to estimate odds ratios (ORs) with 95% confidence intervals (CIs) for VTE according to major surgery and after adjustment for other VTE triggers.

**Results** Surgery was registered in 85 of the 531 (16.0%) hazard periods and in 38 of the 2,124 (1.8%) control periods, yielding an OR for VTE of 11.40 (95% CI: 7.42–17.51). The OR decreased to 4.10 (95% CI: 2.40–6.94) after adjustment for immobilization and infection and was further attenuated to 3.31 (95% CI: 1.83–5.96) when additionally adjusted for trauma, blood transfusion, and central venous catheter. In a mediation analysis, 51.4% (95% CI: 35.5–79.7%) of the effect of surgery on VTE risk could be mediated through immobilization and infection.

**Conclusions** Major surgery was a trigger for VTE, but the association between surgery and VTE risk was in part explained by other VTE triggers often coexisting with surgery, particularly immobilization and infection.

## Introduction

Venous thromboembolism (VTE), encompassing deep vein thrombosis (DVT) and pulmonary embolism (PE), is a common and multicausal disease, occurring as the result of an interplay between environmental and genetic risk factors. 1 Surgery, along with an advancing age, obesity, and cancer, is a major risk factor for VTE. 2 In population-based cohort studies, 15–24% of incident VTEs are associated with surgery. 3 4 As the proportion of elderly in the population is increasing, 5 people at an advancing age with multiple comorbidities and increased susceptibility to complications are more likely to undergo major surgical procedures, which makes the pathophysiology of the relationship between surgery and VTE even more complex.

The mechanism of VTE risk after surgery involves several coexisting factors related to patient demographics and clinical characteristics, the surgical procedure, type of anesthesia, and postoperative complications. 6 7 8 For instance, the VTE risk is particularly high after certain types of surgery, including vascular, orthopaedic, and neurologic procedures. 9 Tissue and blood vessel manipulation and dissection during surgery may trigger activation of blood coagulation and inflammatory responses resulting in a prothrombotic state. 10 11 12 Further, patients may be subjected to prolonged hospitalization and immobilization after surgery, which in turn increase the VTE risk. 13 14 Notably, immobilization has been consistently associated with VTE risk in surgical patients, 15 16 17 18 and infection complications in the postoperative period (e.g., pneumonia) are also well-recognized strong risk factors for VTE in these patients. 6 19 20 21 Other VTE risk factors can also contribute to thrombosis risk in surgical patients, such as major trauma, red blood cell (RBC) transfusion, and central venous catheterization (CVC). 22 23

Confounding is one of the most challenging methodological limitations in observational studies and may be particularly difficult to take into account when assessing the association between surgery and VTE given the multiple factors related to both the exposure (i.e., surgical procedure) and outcome (i.e., VTE). Confounding could be partially addressed with the use of a case-crossover design. Because participants serve as their own controls in the case-crossover study, all potential fixed confounders (e.g., comorbidities, anthropometric, and genetic factors) are largely controlled for through the study design. 24

The role of surgery as a trigger for VTE has been scarcely investigated in case-crossover studies. 23 25 We therefore conceived a case-crossover study derived from the general population to investigate the impact of major surgery as a trigger for incident VTE and to explore to what extent other concomitant VTE triggers, including immobilization and acute infection, could account for the VTE risk in surgical patients.

## Materials and Methods

### Study Population

The study participants were recruited from the Tromsø Study, which is a population-based cohort with repeated health surveys of the residents of Tromsø, Norway. 26 The fourth survey (Tromsø 4) conducted in 1994 to 1995 served as the source population for the present study, where 27,158 individuals aged ≥25 years participated (77% of those invited). The participants were followed from the date of inclusion in Tromsø 4 (1994–1995) until December 31, 2012. All potential first lifetime VTE events were identified using the hospital discharge diagnosis registry, the autopsy registry, and the radiology procedure registry at the University Hospital of North Norway, which is the only provider of hospital care in the Tromsø region. Validation of each VTE event was performed through extensive review of medical records. A VTE was confirmed if signs and symptoms of DVT or PE were combined with objective confirmation by radiological methods, resulting in treatment initiation. 27 A total of 707 individuals experienced an incident VTE event during the follow-up period (1994–2012). The study was approved by the Regional Committee of Research Medical and Health Ethics, and all participants gave their informed written consent.

### Study Design

**Fig. 1 Case-crossover study design.**
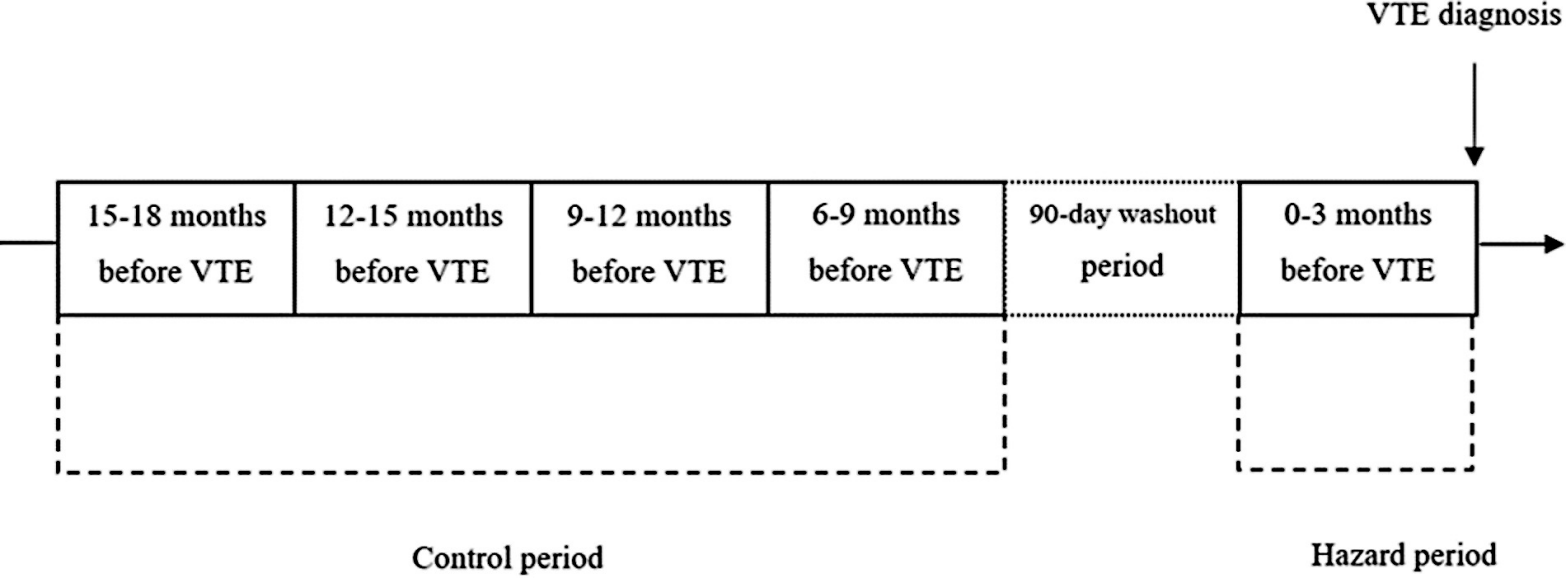
The hazard period was defined as the 90-day period prior to the VTE event. Exposures occurring in the hazard period were compared with exposures occurring during the four previous 90-day control periods. In order to avoid carry-over effects, a 90-day washout period was introduced between the control periods and the hazard period. VTE, venous thromboembolism.

A case-crossover design was applied to investigate the association between major surgery and VTE. In this design, only individuals who have experienced the outcome of interest are included. 24 We defined the 90 days preceding the date of the incident VTE as the hazard period (i.e., risk period), and four consecutive 90-day periods prior to the hazard period as the control periods, as previously described. 13 14 28 29 To avoid potential carry-over effects, a 90-day washout period was introduced between the hazard and control periods ( Fig. 1 ). Exposures in the hazard period were compared with exposures occurring during the four previous 90-day control periods. The rationale behind the use of 90-day periods is based on the current definition of provoking factors for VTE. 30

The case-crossover design is suitable to investigate the effects of transient exposures (e.g., surgery) on acute outcomes (e.g., VTE). 24 We excluded subjects with active cancer at the time of VTE diagnosis ( *n* = 176), because cancer does not necessarily act as a transient exposure or risk factor for VTE, since cancer progression may change an individual VTE risk even within a short time period, which could introduce confounding. Therefore, our final analyses included 531 cancer-free VTE patients.

To conceive the case-crossover study, trained medical personnel systematically evaluated the hospital medical records for each VTE case and recorded potential VTE triggers, in addition to diagnostic procedures, surgical and medical treatment, laboratory tests and diagnoses occurring during hospital admissions, day care, and outpatient clinic visits in any of the control or hazard periods. 13 14 28 29 In this study, there was no access to medical records from general practice.

### Definition of VTE Triggers

A transient risk factor, or trigger, was defined as a risk factor present in the hazard period, i.e., in the 90 days prior to the VTE event, and/or in any of the four preceding 90-day control periods. 13 14 28 29 If an exposure occurred over several days, it was considered to have occurred if any of the days of the exposure fell within the specified 90-day time period. In this case-crossover study, major surgery was registered for operations in organs within the chest, abdomen, pelvic cavity and cranium, and also for hip and knee operations. Minor surgeries were not included and were defined as procedures requiring less than 30 minutes of general anesthesia. 30 31 32

The other triggers were recorded as previously described. 13 14 28 29 In short, immobilization was defined if one of the following factors was present: bedrest for 3 days or more, ECOG (Eastern Cooperative Oncology Group) score of 4, or other immobilizing factors addressed in the patient medical record (e.g., confinement to wheelchair). Infection was recorded if an acute infection was observed in the medical records by a physician and included both hospital-acquired infections and community-acquired infections leading to hospital admission. Infections were registered as respiratory tract infection, urinary tract infection, and other infections. The other VTE triggers, i.e., trauma, RBC transfusion, and CVC, were recorded if noted in the medical record.

### Statistical Analysis

Statistical analyses were carried out using STATA version 16.1 (Stata Corporation, College Station, Texas, United States). Odds ratios (ORs) for VTE were estimated using conditional logistic regression with 95% confidence intervals (CIs) according to the presence of major surgery in the hazard and control periods. The crude association between surgery and VTE in model 1 was adjusted for immobilization and acute infection in model 2, with the addition of the other VTE triggers (i.e., trauma, RBC transfusion, and CVC) to model 3. We also performed subgroup analyses for DVT and PE (with or without concomitant DVT).

Under the assumption that immobilization and infection were complications following surgery, a mediation analysis was performed to quantify the potential of these two triggers to mediate the effect of surgery on VTE risk, using the Karlson–Holm–Breen (KHB) method, which has been extensively described elsewhere. 33 In short, the KHB method allows decomposition of the total effect of the exposure on the outcome into direct and indirect (i.e., mediating) effects. The mediation analysis was carried out with major surgery as the exposure, overall VTE as the outcome, and immobilization and infection as the potential mediators, with adjustment for trauma, RBC transfusion, and CVC. Bootstrapping technique with 10,000 resamples was used to calculate the 95% CIs for mediation percentages estimated by the KHB method.

## Results

The baseline characteristics and the distribution of VTE triggers in the hazard and control periods are shown in Table 1 . Among the 531 cancer-free VTE patients, there were 302 DVTs (56.9%) and 229 PEs (43.1%), the median age at VTE diagnosis was 68 years, and 54% of the participants were women. All triggers of interest occurred more frequently in the hazard period than in the control periods. For instance, major surgery occurred in 85 of the 531 hazard periods (16.0%) and in only 38 of the 2,124 control periods (1.8%). Of note, thromboprophylaxis with low-molecular-weight heparin (LMWH) was prescribed more often in the hazard period (93/531, 17.5%) than in the control periods (42/2,124, 2.0%).

**Table 1:** Baseline characteristics of study participants and distribution of venous thromboembolism (VTE) triggers

*Characteristics*	*At VTE diagnosis (n = 531)*	
Median age, y ± SD	68 ± 14	
Female sex, *n* (%)	287 (54.0)	
Deep vein thrombosis, *n* (%)	302 (56.9)	
Pulmonary embolism a , *n* (%)	229 (43.1)	
*VTE triggers*	*Hazard period (n = 531)*	*Control periods (n = 2,124)*
Major surgery, *n* (%)	85 (16.0)	38 (1.8)
Immobilization b , *n* (%)	152 (28.6)	37 (1.7)
Infection, *n* (%)	166 (31.3)	62 (2.9)
Red blood cell transfusion, *n* (%)	34 (6.4)	12 (0.6)
Trauma, *n* (%)	64 (12.1)	19 (0.9)
Central venous catheterization, *n* (%)	23 (4.3)	5 (0.2)

Abbreviations: SD, standard deviation; VTE, venous thromboembolism.

a Pulmonary embolism with or without concomitant deep vein thrombosis.

b Defined as bed rest >3 days, Eastern Cooperative Oncology Group (ECOG) score of 4, or other immobilizing factors specifically recorded.

The frequency of major surgery in the hazard and control periods and the corresponding ORs for overall VTE, DVT, and PE are displayed in Table 2 . In crude analysis (model 1), the OR for VTE after major surgery was 11.40 (95% CI: 7.42–17.51). After adjustment for immobilization and infection in model 2, the OR decreased to 4.10 (95% CI: 2.40–6.94) and was further attenuated to 3.31 (95% CI: 1.83–5.96) in model 3 with additional adjustment for trauma, RBC transfusion, and CVC. When immobilization and infection were added separately to the regression models, the association between major surgery and VTE was more pronouncedly attenuated when adjusted for immobilization only (OR: 6.11, 95% CI: 3.75–9.94) as compared with infection only (OR: 7.27, 95% CI: 4.51–11.74). In the subgroups, the crude ORs according to the presence of surgery were 13.54 (95% CI: 7.47–24.53) for DVT and 9.25 (95% CI: 4.95–17.28) for PE. Similar to overall VTE, adjustment for other VTE triggers (models 2 and 3) had a considerable impact on risk estimates, particularly for PE.

**Table 2:** Distribution of major surgery in the hazard and control periods and odds ratio for overall venous thromboembolism (VTE), deep vein thrombosis (DVT), and pulmonary embolism (PE)

	Hazard period, *n* (%)	Control periods, *n* (%)	Model 1, OR (95% CI)	Model 2, OR (95% CI)	Model 3, OR (95% CI)
Overall VTE	*n* = 531	*n* = 2,124			
Major surgery	85 (16.0)	38 (1.8)	11.40 (7.42–17.51)	4.10 (2.40–6.94)	3.31 (1.83–5.96)
DVT	*n* = 302	*n* = 1,208			
Major surgery	51 (16.9)	21 (1.7)	13.54 (7.47–24.53)	5.26 (2.60–10.67)	5.41 (2.49–11.76)
PE	*n* = 229	*n* = 916			
Major surgery	34 (14.8)	17 (1.9)	9.25 (4.95–17.28)	2.89 (1.27–6.57)	1.55 (0.61–3.96)

Abbreviations: CI, confidence interval; OR, odds ratio.

Note: Model 1: unadjusted OR.

Model 2: adjusted for immobilization and infection.

Model 3: adjusted for immobilization, infection, trauma, red blood cell transfusion, and central venous catheterization.

To analyze the magnitude of the potential mediating effects of immobilization and infection on the relationship between surgery and overall VTE, a mediation analysis was carried out ( Table 3 ). The KHB method estimated that 51.4% (95% CI: 35.5–79.7%) of the association between surgery and VTE risk was due to a mediating effect (i.e., indirect effect) acting via immobilization and infection, in analyses adjusted for trauma, RBC transfusion, and CVC. With regard to the mediating effect, 65.4% (95% CI: 48.0–80.7%) was attributable to immobilization and 34.6% (95% CI: 19.3–52.0%) to infection.

**Table 3:** Mediation analysis of the association between major surgery and overall venous thromboembolism (VTE) according to the Karlson–Holm–Breen (KHB) method

Decomposition of logistic regression	Coefficient	Standard error	Mediation percentage (95% CI)
Total effect	2.47	0.32	
Direct effect	1.20	0.30	
Indirect effect	1.27	0.24	51.4% (35.5–79.7%)
*Mediation through*			
Immobilization	0.83		65.4% (48.0–80.7%)
Infection	0.44		34.6% (19.3–52.0%)

Abbreviation: CI, confidence interval.

Note: In the KHB method, the total effect of major surgery on VTE risk is decomposed into direct and indirect effects. The mediation through immobilization and infection corresponds to the indirect effect. The mediation analysis was adjusted for other VTE triggers, i.e., trauma, red blood cell transfusion, and central venous catheterization. The 95% CIs were calculated by bootstrapping with 10,000 resamples.

## Discussion

The results from this case-crossover study confirm that surgery is a major trigger for incident VTE. However, the association between surgery and VTE was attenuated after adjustments for immobilization and acute infection, and a further decrease in risk estimates was noted with additional adjustment for other VTE triggers (i.e., trauma, RBC transfusion, and CVC). In a mediation analysis, about 50% of the effect of surgery on the risk of VTE was mediated by immobilization and infection. Thus, our findings indicate that other VTE triggers frequently related to surgical procedures, particularly immobilization and infection, in part contribute to thrombosis risk after a major surgery.

To our knowledge, this is the largest case-crossover study that has been conceived to investigate the role of major surgery as a VTE trigger while taking other concomitant VTE triggers into account. Only a few studies have investigated the association between surgery and VTE using a case-crossover design. 23 25 The first study was conducted in the United States by Rogers et al 23 and involved 399 VTE events identified by the use of International Classification of Diseases (ICD) codes among participants of the Health and Retirement Study (≥51 years of age), whose data were linked to the Medicare service. Similar to our findings, the association between major surgery and VTE was attenuated after adjustment for other VTE triggers, including immobilization, infection, blood transfusion, and CVC. In a more recent study based on a large medical database in France, Caron et al investigated the duration and magnitude of the postoperative thrombosis risk among 60,703 cancer-free patients aged 45 to 64 years with a PE diagnosis identified by ICD codes between 2007 and 2014. 25 Even though the risk of PE was highest during the first 6 weeks after surgery, risk estimates remained substantially elevated for at least 12 weeks after all types of surgery (i.e. gastrointestinal, gynecological, vascular, and orthopaedic surgical procedures). However, the association between surgery and PE was not adjusted for other VTE triggers commonly associated with surgery. Additionally, in both the American and French case-crossover studies, 23 25 the assessment of the VTE events was carried out using ICD codes, which could have potentially led to some degree of misclassification.

In this study, the association between major surgery and VTE risk was attenuated when taking other VTE triggers into account, particularly immobilization and infection. Here, immobilization and acute infection appeared to mediate approximately 50% of the effect of major surgery on VTE risk, with almost two-thirds (65%) of the mediating effect being attributable to immobilization. Indeed, immobilization leading to venous stasis is a major contributor to VTE risk in the postoperative setting 15 16 17 18 and emphasizes that early mobilization after surgery along with evidence-based thromboprophylaxis is key to reduce the incidence of VTE. 34 35 Acute infection, including surgical site infection, is a common complication after surgery and is associated with longer postoperative hospital stays, additional surgical procedures, treatment in intensive care units, and higher mortality. 36 Additionally, in cohort studies of patients undergoing several types of major surgery, infections, including pneumonia, urinary tract infection, and surgical site infection have been consistently associated with increased risk of VTE in the postoperative period. 6 19 20 21

It is noteworthy that even after adjustment for all VTE triggers, surgery was still associated with a threefold increased risk of VTE. In a case-crossover study, all fixed confounders are largely controlled for through the design and are therefore unlikely to influence the results. 24 The remaining VTE risk could be due to those factors that are direct consequences of the surgical procedure, including tissue and blood vessel manipulation. This may cause significant endothelial damage, activation of blood coagulation, and an increased inflammatory response. 10 11 12 Inflammation might accentuate the hypercoagulable state by promoting endothelial dysfunction as well as activation of coagulation. 10 Surgical interventions have also been reported to be associated with transient platelet activation and generation of cell-derived microparticles with procoagulant activity. 10 37 38 Hence, the surgical procedure itself presumably contributes to the VTE risk through the coexistence of multiple pathophysiological pathways.

The strengths of this study include the case-crossover design, which allows the investigation of the effects of transient exposures (such as surgery) on acute outcomes (like VTE), while controlling for potential fixed confounders as the participants serve as their own controls. 24 This case-crossover study was representative of the general, cancer-free VTE population, since the VTE events were derived from a large population-based cohort study with a wide age distribution. Importantly, all the VTE events, including DVT and PE, were validated applying objective criteria. There are some limitations that merit attention. Information on exposure to VTE triggers was obtained without assessing the temporal sequence between them within each 90-day period. Thus, the mediation analysis was conducted under the assumption that surgery was present before immobilization, infection, and the other VTE triggers in each period. Although unlikely, we cannot rule out that in some cases, surgery could have taken place after the exposure to the other VTE triggers. Therefore, caution is needed when interpreting the results from the mediation analysis. Residual confounding cannot be completely excluded due to unmeasured or unknown transient risk factors that could have influenced the association between surgery and VTE. Thromboprophylaxis with LMWH was prescribed more often in the hazard period (17.5%) than in the control periods (2.0%), most likely due to confounding by indication, i.e., patients regarded to be at a high risk of developing VTE during the hazard period were those who most likely had the indication of thromboprophylaxis. Consequently, we could not adjust our analyses for thromboprophylaxis as this would have introduced bias. It is worth noting that seasonality of infection could potentially affect the association between infection and VTE. However, as demonstrated in our previous report using the same case-crossover study, 28 results remained essentially similar in sensitivity analyses restricted to comparison of the hazard period with the control period that occurred 12 to 15 months before VTE (i.e., the control period that represents exactly the same season as the hazard period). Hence, seasonality of infection is unlikely to have any substantial impact on our findings regarding the association between infection and VTE. Finally, because of the limitations regarding the sample size, we could not conduct subgroup analyses according to the type of surgery.

In conclusion, major surgery was a trigger for incident VTE in this case-crossover study. Our findings indicate that other VTE triggers often coexisting with surgery, particularly immobilization and infection, are important contributors to the VTE risk after major surgical procedures.
